# The restless brain: how intrinsic activity organizes brain function

**DOI:** 10.1098/rstb.2014.0172

**Published:** 2015-05-19

**Authors:** Marcus E. Raichle

**Affiliations:** Mallinckrodt Institute of Radiology, Washington University School of Medicine, 4525 Scott Avenue, Room 2116, St Louis, MO 63110, USA

**Keywords:** aerobic glycolysis, functional connectivity, local field potentials, neoteny, resting state, slow cortical potentials

## Abstract

Traditionally studies of brain function have focused on task-evoked responses. By their very nature such experiments tacitly encourage a reflexive view of brain function. While such an approach has been remarkably productive at all levels of neuroscience, it ignores the alternative possibility that brain functions are mainly intrinsic and ongoing, involving information processing for interpreting, responding to and predicting environmental demands. I suggest that the latter view best captures the essence of brain function, a position that accords well with the allocation of the brain's energy resources, its limited access to sensory information and a dynamic, intrinsic functional organization. The nature of this intrinsic activity, which exhibits a surprising level of organization with dimensions of both space and time, is revealed in the ongoing activity of the brain and its metabolism. As we look to the future, understanding the nature of this intrinsic activity will require integrating knowledge from cognitive and systems neuroscience with cellular and molecular neuroscience where ion channels, receptors, components of signal transduction and metabolic pathways are all in a constant state of flux. The reward for doing so will be a much better understanding of human behaviour in health and disease.

## 1. Introduction

Since the introduction of electroencephalography (EEG) in humans by Hans Berger in 1929 [1] (for an English translation of this important work see [2]), it has been clear that ongoing spontaneous electrical activity is a prominent feature of the brain of every species in which it has been studied including humans. In referring to the spontaneous activity in the human EEG, Berger rhetorically asked [2, pp. 562–563] ‘Is it possible to demonstrate the influence of intellectual work upon the human electroencephalogram, insofar as it has been reported here?’ He concluded that: ‘Of course, one should not at first entertain too high hopes with regard to this, because mental work, as I explained elsewhere, adds only a small increment to the cortical work which is going on continuously and not only in the waking state’. Consistent with Berger's prediction it has subsequently been shown that extensive averaging of the EEG is necessary to attenuate if not eliminate this seemingly random, ongoing activity in order to see event-related potentials (ERPs).

Despite the implication of Berger's early work showing that substantial activity is always present and should be accounted for [1], the motivating focus of neuroscience research has been on event-related activity (i.e. the brain is reflexive, primarily driven by the momentary demands of the environment). From a practical point of view, this is not surprising because experiments designed to measure brain responses to controlled stimuli and carefully designed tasks can be rigorously controlled and the results of such experiments measured with great precision, whereas evaluating the behavioural relevance of intrinsic activity (i.e. ongoing neural and metabolic activity which may or may not be directly associated with subjects' performance) can be an elusive enterprise. Unfortunately, the success of studying evoked activity has caused us to lose sight of the possibility that our experiments reveal only a small fraction of the actual functional activity performed by our brain.

In this essay, I review the evidence that persuades me of the importance of intrinsic activity and then briefly survey the material presently available regarding its properties and functions. The story is incomplete but rich with opportunities for future research that will be most productive if conducted in a climate of mutual respect for different levels of analysis.

## 2. Adjudicating the merits of intrinsic activity

### (a) Cost

One of the most persuasive arguments for the importance of intrinsic activity emerges from a consideration of its relative cost in terms of brain energy consumption. In the average adult human, the brain represents about 2% of the total body weight yet it accounts for 20% of all the energy consumed [3,4], 10 times that predicted by its weight alone.

Relative to this very high rate of ongoing energy consumption in the resting state, the additional energy consumption associated with changes in brain activity is remarkably small, often less than 5% of the baseline level of activity [5]. From these data, it is clear that the brain's enormous energy consumption (something I have dubbed the brain's *dark energy* [6,7]^1^) is little affected by task performance, an observation first made more than 50 years ago by Louis Sokoloff, Seymour Kety and their colleagues [8] but rarely cited (see also [4] for an interesting evolutionary perspective).

What is the nature of this ongoing intrinsic activity that commands such a large amount of the brain's energy resources? Assessments of brain energy budget using a variety of approaches (for review, see [5]) would suggest that 60–80% of overall brain energy consumption is devoted to *spike-generated* glutamate cycling and, hence, neural signalling processes involving principal cells. The basis for this estimate, however, should be viewed with caution for several reasons. First, it is important to realize that most of the ongoing electrical activity of the neocortex is, in fact, subthreshold depolarizations rather than action potential firing ([9], see also [10]). Second, early estimates of the cost of spikes fell far short of explaining the cost of brain function [11]. Also, current estimates leave for future consideration the demands placed on the brain's energy budget by the activity of inhibitory interneurons [12–17], astrocytes [18,19] and other supporting cells [20].

Furthermore, it is important to emphasize that biosynthesis may be a significant contributor to the cost of brain function [21]. Eve Marder has described the situation nicely [22, p. 563]: ‘Humans and other long-lived animals … have neurons that live and function for decades. By contrast, ion channel proteins, synaptic receptors and the components of signal transduction pathways are constantly turning over in the membrane and being replaced, with half-lives of minutes, hours, days or weeks. Therefore, each neuron is constantly rebuilding itself from its constituent proteins, using all of the molecular and biochemical machinery of the cell. This allows for plastic changes in development and learning but also poses the problem of how stable neuronal function is maintained … ’ As Locasale & Cantley [21] have pointed out, basal cellular maintenance of the type Eve Marder describes is very costly, something probably underestimated [23]. This is a subject to which I will return later in this essay (see Intrinsic activity and metabolism).

### (b) Sensory information

Complementary insight on the importance of intrinsic activity comes from a consideration of sensory information. It may surprise some to learn that visual information is significantly compressed as it passes from the eye to the visual cortex [24,25]. Thus, of the information available from the environment, only about 10^10^ bits s^−1^ are deposited in the retina. Because of a limited number of axons in the optic nerves (approx. 1 million axons in each) only 10^6^ bits s^−1^ leave the retina and only 10^4^ make it to layer IV of V1. These data clearly leave the impression that visual cortex receives a very compressed representation of the world, a subject of more than passing interest to those interested in the processing of visual information [26]. Parenthetically, it should be noted that estimates of the bandwidth of conscious awareness itself (i.e. what we ‘see’) are in the range of 100 bits s^−1^ or less [25].

Reinforcing this impression of the brain's ‘isolation’ is the fact that the number of synapses in the lateral geniculate nucleus of the thalamus and in layer IV of primary visual cortex devoted to incoming visual information is less than 10% of the total number of synapses in both locations [27]. Various proposals have been made concerning the interpretation of these anatomical data [28,29] but the fact remains that the brain must interpret, respond to and even predict environmental demands from seemingly impoverished data. An explanation for its success in doing so must lie in significant measure with intrinsic brain processes that link representations residing broadly within brain systems to incoming sensory information [30].

Vernon Mountcastle, one of the preeminent neurophysiologists of the twentieth century, summed up the situation nicely: ‘Each of us believes himself to live directly within the world that surrounds him, to sense its objects and events precisely, and to live in real and current time. I assert that these are perceptual illusions. Sensation is an abstraction, not a replication, of the real world’ [31].

## 3. The organization of intrinsic activity

Important insights into the organization of intrinsic activity have come from two perspectives: a top–down approach using brain imaging with PET and fMRI as well as genetics in normal humans and electrocorticography in selected patients; and a bottom–up approach using laboratory animals and more invasive, high-resolution (spatial and temporal) studies employing neurophysiological as well as optical imaging techniques. Together a picture of the dynamic organization of intrinsic activity emerges that is remarkably complementary across these levels of analysis.

### (a) Top–down view: activity decreases from a resting state^2^

By the early 1980s, PET began to receive serious attention as a potential functional neuroimaging device in human subjects [32]. The study of human cognition with neuroimaging was aided greatly by the involvement of cognitive psychologists in the 1980s whose experimental strategies for dissecting human behaviours fitted well with the emerging capabilities of functional brain imaging [33]. This strategy, involving the careful selection of task and control states, was a major contribution of cognitive psychology to the emerging field of cognitive neuroscience. This approach, in various forms, has dominated the cognitive neuroscience agenda ever since with remarkably productive results (e.g. [34]).

**Figure 1.**
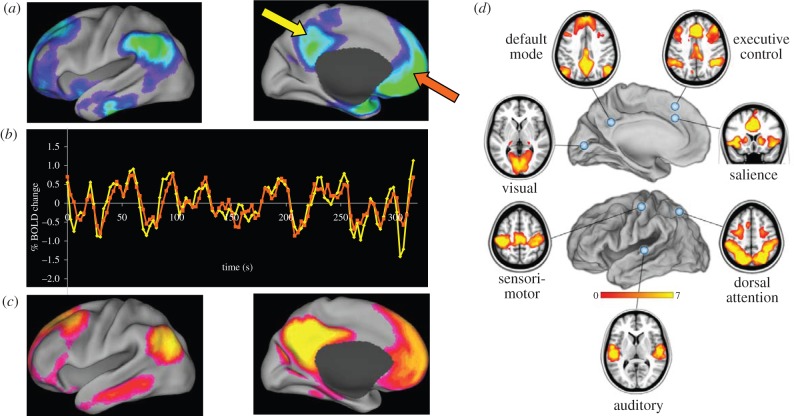
Performance of a wide variety of tasks has called attention to a group of brain areas (*a*) that decrease their activity during task performance. These areas are often referred to as the brain's default mode network (DMN). If one records the spontaneous fMRI BOLD signal activity in these areas in the resting state (arrows, *a*) what emerges is a remarkable similarity in the behaviour of the signals between areas (*b*). Using these fluctuations to analyse the network as a whole reveals a level of functional organization in the ongoing intrinsic activity of the brain (*c*) that parallels that seen in the task-related activity decreases (*a*). Analyses of other brain systems (*d*) reveal similar levels of functional organization that exist in concert with their subcortical connections (not shown). Elements of this figure were adapted from [36,37] with permission.

One of the guiding principles of cognitive psychology was that a control state must explicitly contain all of the elements of the associated task state other than the one element of interest (e.g. seeing a word versus reading the same word). Using a control state of rest would clearly seem to violate that principle. Despite our commitment to the strategies of cognitive psychology in our experiments we routinely obtained resting state scans in all of our experiments, which was a carry-over habit from experiments involving simple sensory stimuli [35] where the control state was simply the absence of the stimulus (i.e. a resting state^2^). At some point in our work, and I do not recall the motivation, I began to look at the resting state scans minus the task scans. What immediately caught my attention was the fact that regardless of the task under investigation, activity decreases were clearly present and almost always included the posterior cingulate and the adjacent precuneus (figure 1*a*). Initially puzzled by the meaning of this observation, I began collecting examples from our work and placed them in a folder which I labelled MMPA for mystery medial parietal area.

The first formal characterization of task-induced activity decreases from a resting state was a meta-analysis of nine PET studies involving 134 subjects by my colleague Gordon Shulman [38]. This study generated an iconic image of a network of cortical areas that decreased their activity during the performance of a variety of attention-demanding, largely non-self-referential tasks (figure 1*a*). The unique identity of this network was confirmed a short time later by others [39,40] with similar observations which are now an everyday occurrence in laboratories worldwide as investigators seek to understand its role in brain function. This network has been dubbed the brain's *default mode network* (DMN) by Greicius *et al*. [41] after our formal description of its unique features [42]. Subsequent work by us and others (summarized in [43]) has established the DMN as an important functional component of the intrinsic activity of the human brain as well as in non-human primates [44] and rodents [45,46].

It should be noted that other more task-specific deactivations had been noted by us and others [47–53], consistent with our more general idea that a default mode of brain function [42,54] is broadly based across all brain systems (a hypothesis that was to receive substantial support from functional studies of the brain's resting state^2^ [36,37]).

The discovery of the DMN made apparent the need for additional ways to study the large-scale intrinsic, functional organization of the brain. A major step forward was the discovery that this large-scale network organization, including but not limited to the DMN, could be revealed by the study of patterns of spatial coherence in the spontaneous fluctuations (i.e. noise) of the fMRI blood-oxygen level dependent (BOLD) signal.

### (b) Top–down view: spontaneous fluctuation in the fMRI signal

A prominent feature of fMRI is the noise in the raw, resting state BOLD signal (figure 1*b*). For many years, this prompted researchers to average their data to increase signal and reduce noise. As first shown by Biswal *et al*. [55] in the human somatomotor system, this ‘noise’ exhibits strong patterns of coherence within well-known brain systems.

The significance of this observation was brought forcefully to our attention when Greicius *et al*. [41] looked at the patterns of coherence in the DMN elicited by placing a region of interest in either the posterior cingulate cortex (yellow arrow, figure 1*a*) or the ventral medial prefrontal cortex (orange arrow, figure 1*a*). The resulting time-activity curves (figure 1*b*) reflected a pattern of coherence within the entire DMN (figure 1*c*). Similar patterns of resting state coherence have now been documented in most cortical systems in the human brain (figure 1*d*) as well as their subcortical connections [56–58].

A number of additional observations made about these surprising patterns of spatial coherence are of interest. First, they appear to transcend levels of consciousness, being present under anaesthesia in humans [59], monkeys [44], rats [45] and mice [46] and also during sleep in humans [60–62]. These observations make it unlikely that the patterns of coherence and the intrinsic activity they represent are primarily the result of unconstrained, conscious cognition (i.e. mind-wandering or day dreaming [63]).

Second, while resting state patterns of coherence in fMRI do respect patterns of anatomical connectivity in both the monkey [44] and human brain [56], it is clear that they are not constrained by these anatomical connections. Thus, the absence of monosynaptic connections between brain areas (e.g. right and left primary visual cortex [44]) does not preclude the existence of functional connectivity as expressed in the maps of resting state coherence.

Third, relationships thus far uncovered among the resting state networks (figure 1*d*) reveal a distinct hierarchy (e.g. [64–67]) that places the DMN in a central role within the organization of the brain's intrinsic functional network structure. Furthermore, these relationships are not static. For example, the DMN and the dorsal attention network (DAN; figure 1*d*) are anti-correlated in the resting state [68], a relationship noted during the performance of novel, attention-demanding, non-self-referential tasks in which the DMN decreases its activity while the DAN is increased. Interestingly, this resting state relationship has received confirmation in direct electrophysiological measurements in laboratory animals [69]. Furthermore, the resting state relationship between the DMN and medial temporal structures varies diurnally, absent in the morning but present in the evening [70], probably reflecting changes in brain organization related to the accrual of new knowledge while awake.

Finally, spontaneous fluctuations in the BOLD signal contribute significantly to both variability in evoked signals [71] and variability in the associated behaviour [72], observations that were anticipated by others pursuing the neurophysiology more directly ([73–75], also see below).

### (c) Bottom–up view: organization at the cellular level

Occurring in parallel but rarely interacting at a conceptual level with the ‘top–down view’ of intrinsic activity has been *in vivo* [74,76–78] and slice work [79,80] primarily on the sensory cortices (but also see [81]) in laboratory animals, employing conventional electrode recordings as well as voltage-sensitive dye and calcium imaging. Several interesting themes emerge from this very important work.

**Figure 2.**
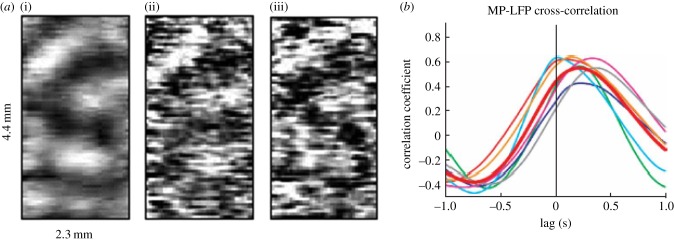
Measurements in laboratory animals provide a more detailed picture of intrinsic activity at the cellular level complementing nicely data from humans. (*a*) Voltage-sensitive dye imaging of spontaneous and evoked activity in the visual cortex of the anaesthetized cat: (i) an averaged orientation map using full-field gratings of vertical orientation; (ii) a map obtained in a single frame from a spontaneous recording session and (iii) a single frame from an evoked session. Spontaneous and evoked activities are remarkably similar as noted in figure 1 as well. Adapted from [76] with permission. (*b*) This graph from [84] provides a very nice demonstration of how the membrane potentials (i.e. UDS) of CA1 hippocampal interneurons are spontaneously phase-locked to the LFPs of parietal cortex neurons in the mouse, suggesting a mechanism by which a systems level organization (e.g. as in figure 1) might arise (used with permission). The latency shown in this figure (i.e. ± 1 s) is remarkably similar to that recently shown by us to exist within and among systems in the human brain [85].

In a series of papers on the cat visual cortex using a combination of electrode recording and voltage-sensitive dyes from the Weizmann Institute beginning in 1995 [74,76,82,83], it was shown that the magnitude of ongoing intrinsic activity was the same as evoked activity, and that the two interacted strongly with the intrinsic activity contributing significantly to the variability in evoked activity, confirming an observation first made by George Bishop in 1933 [73]. And, even in the absence of stimuli, cortical representations of visual attributes emerged from the ongoing spontaneous activity (figure 2*a*, [76,86]). These authors also concluded that a significant fraction of the intrinsic activity represented subthreshold activity within dendrites, a recurrent theme in later publications (e.g. [77]). Elegant replications and extensions of this work have been contributed by others [77–80].

In summary, combining a bottom–up and a top–down view of intrinsic activity reveals common themes at the cellular level and full brain systems level. At a very local level, at least in sensory cortices, intrinsic activity is organized into the cortical representations of anticipated sensory attributes. At the full brain level, in the absence of any overt activity, intrinsic activity is organized into systems well known for their participation in the full range of overt behaviours. This organization is clearly hierarchical from the local to the systems level and consistent with the hypothesis that the brain maintains a state of preparedness in anticipation of the demands placed upon it while awake. But considering the fact that intrinsic, spontaneous activity persists during sleep we should be mindful of its potential role in development and plasticity [87,88].

## 4. The neurophysiology of intrinsic activity

There has been an active effort to ascertain the electrical correlates of the fMRI BOLD signal for some time (for summaries of this work from different perspectives, see [5,89,90]). The conclusion to be drawn from this work as I see it is that the fMRI BOLD signal is best correlated with local field potentials (LFPs), that is, the complex, subthreshold signals arising from the integrated electrical activity in pre- and postsynaptic terminals of the brain. The research shows further that the spontaneous fluctuations in the BOLD signal are best correlated with LFP activity in the range of *slow cortical potentials* (SCPs; approx. 0.01–5 Hz [91,92]).

SCPs provide a window on how the brain matches its predictions to changing environmental contingencies. Schroeder & Lakatos [93] view this as one mode of attending in which the phase of the SCPs is shifted to match the predictable patterns of incoming information, a process dubbed phase resetting. As a result, responses are enhanced and performance is improved (see also [75,94–97]). This mode, and it may well be the dominant mode, occurs in a seemingly effortless manner fitting, in a sense, the idea of a default mode of brain function involving the ongoing coordinated activity of all of its systems. It provides a means of connecting the concept of an intrinsic mode of brain function designed to organize information for interpreting, responding to and even predicting environmental events [98,99] to register with the naturally occurring but ever changing regularities unfolding in the environment.

## 5. Relating systems to cells

At the cellular level, spontaneous activity is often discussed in terms of variations in neuronal excitability (e.g. [72,75,82,83]) mediated by spontaneous variations in membrane voltage known as *up and down states* (UDS). The question is whether a similar mechanism underlies the spontaneous fluctuations in the fMRI BOLD signal that gives us resting state maps of functional connectivity (figure 1).

On the basis of this information, we originally thought it reasonable to ask whether spontaneous fluctuations in the fMRI BOLD signal were, in fact, related to UDS (see Supplementary Note 3 in [100]). We concluded at the time that it was unlikely to be the case for two reasons. First, the frequency content of the BOLD signal demonstrates a power spectrum that exhibits power law scaling (for a review, see [101]), whereas UDS have a narrow frequency range that centres around 0.8 Hz. And, second, UDS and their associated LFPs travel across the cortex with latencies of a second or less (figure 2*b*, [84]), whereas fMRI BOLD resting state networks appear spatially stationary. Recent advances in our analysis of spontaneous fluctuations in the fMRI BOLD signal [85], however, suggest a rethinking of that view.

We find that the resting state fMRI BOLD signal contains even more interesting features of the organization of the brain's intrinsic activity than initially thought [85]. The traditional way to examine the correlation structure of intrinsic activity using the fMRI BOLD signal is to average across time. This simple manoeuvre has been surprisingly powerful in identifying patterns of activity that are spatially structured (figure 1*d*), linked to the representation of function and clinically relevant [102]. Importantly, the computational strategies employed in this work [57] make the critical assumption that the activity within networks is exactly synchronous. However, evidence from a variety of sources (for a recent review, see [85]) suggests that intrinsic activity is spatio-temporally structured. We recently explored the latency structure of the spontaneous fluctuations of the fMRI BOLD in detail [85] and found that intrinsic activity propagates orthogonal to conventional resting state networks on a timescale of approximately 1 s, precisely in the range of UP and DOWN states (figure 2*b*) [84]). These findings open up a whole new avenue of investigation involving the temporal as well as spatial structure of intrinsic activity and provide a means of linking activity at the systems level (i.e. the temporal features of the spontaneous fMRI BOLD signal) to that at a cellular level (i.e. changes in excitability or UDS).

## 6. Intrinsic activity and metabolism

Pursuing an understanding of the brain's intrinsic activities needs not stop with the neurophysiology. Understanding the underlying cell biology is also relevant in understanding not only brain imaging signals but also what these signals are actually telling us about brain function (for those interested in an expanded view of this argument, see [5,54,103]). Functional brain imaging studies actually provide some clues as to how this inquiry might proceed.

**Figure 3.**
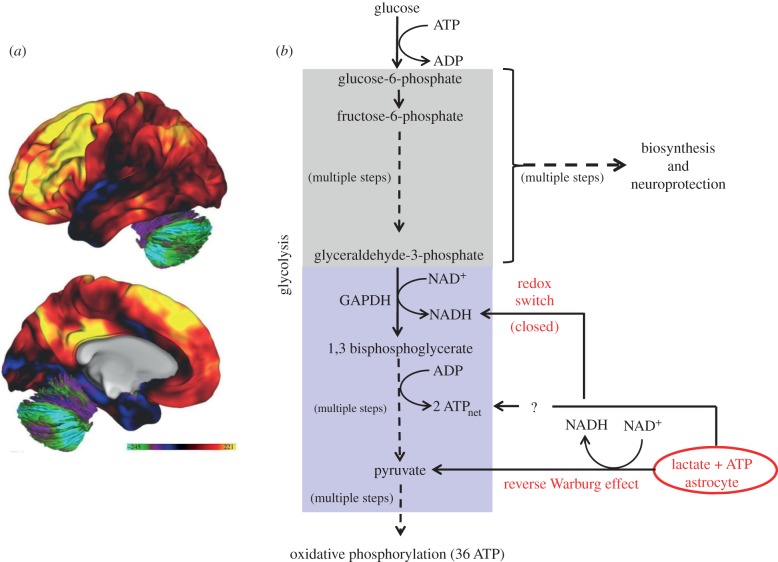
Aerobic glycolysis refers to glycolysis in the presence of oxygen that exceeds that needed for oxidative phosphorylation. (*a*) A map of aerobic glycolysis here illustrated on the lateral and medial surfaces of the human brain in 33 normal young adults [106]. The colour bar is in units of a *glycolytic index,* a quantitative measure of glycolysis [106]. The levels of aerobic glycolysis vary significantly within the brain. Adapted from [103] with permission. (*b*) A very simplified depiction of glycolysis highlighting features discussed in detail in the text. Elements of glycolysis are highlighted by two coloured boxes to denote those elements involved in biosynthesis and neuroprotection (grey) and those involved in energy generation (blue). The diagram is also meant to highlight the symbiotic relationship between astrocytes and neurons which not only involves providing substrate (i.e. lactate) for energy generation via oxidative phosphorylation (reverse Warburg effect) but also, in so doing, how astrocyte lactate alters the redox potential of the neuron (redox switch) to divert neuronal glycolysis into biosynthesis and neuroprotection (i.e. management of reactive oxygen species). Astrocytes also have been shown to regulate UP states through a purinergically mediated mechanism [107]. Because astrocytes release ATP [108] along with lactate, it is attractive to posit regulation of UP states via K_ATP_ channels in the neuron.

I begin from the perspective of the fMRI BOLD signal, whose cell biology helps introduce a somewhat more sophisticated view of the role of the metabolism of intrinsic activity, one that goes beyond a consideration of metabolism solely in terms of energy generation. One of the surprising observations made with PET was that blood flow increases much more than oxygen consumption during task-induced increases in brain activity [104,105]. The practical significance of this observation paved the way for fMRI [32]. However, overlooked by many in discussions of BOLD signal biology have been the task-induced increases in aerobic glycolysis (i.e. glucose metabolized by the brain in excess of that needed for oxidative phosphorylation despite the presence of adequate oxygen; figure 3) that accompany changes in blood flow. These unexpected task-induced increases in aerobic glycolysis actually reflect an increase rather than a de novo appearance of aerobic glycolysis.

Indeed, aerobic glycolysis is present in the normal adult human brain *at rest*, accounting for 12–15% of the glucose metabolized [109,110]. Hence it constitutes an important component of intrinsic activity metabolism [106]. Furthermore, aerobic glycolysis is not distributed uniformly (figure 3*a*). Rather, it exhibits elevated levels in the DMN and adjacent areas of the dorsolateral prefrontal cortex and low levels in the cerebellum and the medial temporal lobes [106].

Taking advantage of the non-uniform distribution of aerobic glycolysis, we compared the regional variance in the resting state fMRI BOLD signal (figure 1*b*) with the regional levels of aerobic glycolysis in the human brain (figure 3*a*) and found them to be highly correlated. Thus, aerobic glycolysis at ‘rest’ is related to dynamic imaging signals that have allowed us to delineate the spatial and temporal components of the brain's functional organization (for a further illustration and discussion, see fig. 6 in [5]). It is important to understand what this might mean with regard to the cellular mechanisms involved.

Through a series of important experiments beginning in the early 1990s (reviewed in [111]), it was established that one source of aerobic glycolysis is the energy demands of the membrane pump Na,K-ATPase in astrocytes [111]. Glutamate is removed from the synapse by uptake into astrocytes in a sodium-dependent process. Sodium must then be removed from the astrocyte by Na,K-ATPase. The energy needed for this process comes from aerobic glycolysis which produces a net 2 ATP per molecule of glucose consumed. One might argue that it is inefficient to fuel such a critical pump by aerobic glycolysis given such a low yield of ATP for each molecule of glucose used. However, the advantage aerobic glycolysis has over oxidative phosphorylation is that the ATP is produced much faster (at least two times faster [112]). Thus, where speed and flexibility is important, such as at an excitatory synapse, one might posit that aerobic glycolysis is the way to go. Regardless of the reason, it is the case that Na,K-ATPase is commonly fueled by aerobic glycolysis in all membrane systems in which it is found [113–116] with lactate as a by-product.

As Pellerin and Magistretti have shown [117], monocarboxylate transporters favour the movement of lactate out of the astrocyte and into the neuron in both the cell body and the postsynaptic density. They further posited that lactate would move from the astrocyte to the neuron to supplement the energy needs of the neuron (figure 3*b*), an idea that generated much controversy [5,118–121]. Unfortunately, these discussions have tended to ignore the elegant details of that relationship. I briefly highlight some of the most interesting features of this symbiotic relationship between neurons and astrocytes.

When lactate enters a neuron its conversion to pyruvate and entry into oxidative phosphorylation captures only part of the story. It is not a simple choice by the neuron to select between glucose and lactate for its energy production but, rather, the challenge for the neuron is to maintain energy production while at the same time increasing the availability of glucose for biosynthesis and neuroprotection. Overlooked by most neuroscientists is one of the important functions performed by glycolysis which is to provide substrate for biosynthesis [21,103,122].

Biosynthesis via glycolysis proceeds largely via the *pentose phosphate pathway* (figure 3*b*), where glucose-derived carbon is used for the synthesis of nucleotides, lipids and proteins. This is important not only for actively proliferating cancer cells, where the role of aerobic glycolysis has been explored in great detail [123], but for the basal turnover and remodelling of neuronal connections in the service of memory and learning (e.g. [103,124,125]). This expanded view of aerobic glycolysis in a symbiotic relationship between neuron and astrocyte has been dubbed the *reverse Warburg effect* [126] (figure 3*b*) in reference to the original work of Otto Warburg on the role of aerobic glycolysis in cell proliferation [127]. This same relationship is seen in axons where the supporting cell is the oligodendrocyte [20].

There is an additional fascinating twist to the *reverse Warburg effect* [126] which involves the redox state of the neuron. When lactate enters the neuron and is converted to pyruvate it shifts the NAD^+^/NADH equilibrium to a more reduced state which turns off glycolysis at a critical step between biosynthesis pathways and energy generation (i.e. the conversation of glyceraldehyde-3-phosphate to 1,3 bisphosphoglycerate mediated by GAPDH; figure 3*b*). This has been dubbed a *redox switch* [128], designed to facilitate glycolysis-mediated biosynthesis in the neuron without sacrificing its mandatory energy requirements which are conveniently supplied by lactate from an adjacent astrocyte. With a potential role for aerobic glycolysis in cellular biosynthesis in the human brain, it is important to ask what evidence we have for this hypothesis.

To pursue the hypothesis put forth above that elevated aerobic glycolysis is associated with biosynthesis, we explored its regional variability in relation to gene expression [103] and found that aerobic glycolysis correlates with the persistence of gene expression typical of infancy (transcriptional neotony). In brain regions with the highest aerobic glycolysis levels (figure 3*a*), we found increased gene expression related to synapse formation and growth. By contrast, regions high in oxidative glucose metabolism express genes related to mitochondria and synaptic transmission. Our results suggest that brain aerobic glycolysis in the resting state supports developmental processes, particularly those required for synapse formation and replacement. Such processes are ongoing in the adult human brain particularly in areas such as the brain's DMN [129].

Consistent with the hypothesis that aerobic glycolysis is important for biosynthesis is the trajectory of human brain metabolism during the first two decades of life (for a review of this literature, see [103]). By age 2, the glucose metabolism of the infant brain has reached adult levels and by the end of the first decade of life it is, remarkably, twice that of the adult (see fig. 2 in [103]). Thirty per cent of glucose use in an average 10 year old is aerobic glycolysis. Levels of glucose metabolism, aerobic glycolysis and oxygen consumption decline to adult levels early in the second decade of life. This time course parallels remarkably that of synaptic proliferation and pruning. Finally, adult levels of synapses appear to be maintained through a dynamic balance between synaptic proliferation and synaptic elimination [129]. Aerobic glycolysis is needed in this situation where constituents are being constantly remodelled in the service of learning and memory.

It should be noted that the hippocampus, long associated with learning and memory, actually has a low level of aerobic glycolysis (figure 3*a*). Further work will obviously be needed to understand the implications of this with regard to the role of the hippocampus and other medial temporal lobe structures in learning and memory. Bringing in insights from metabolism and cell biochemistry will probably be very informative.

It is worth coming back to the spontaneous fluctuations of the fMRI BOLD signal which have provided such important new insights into the organization of the intrinsic activity of the brain (figure 1) as well as the more recent findings of a latency structure within this signal that has the temporal properties of UDS [85] a critical component of the cellular elements of intrinsic activity reviewed earlier (figure 2). A recent paper by Poskanzer & Yuste [107] convincingly shows that astrocytes regulate neuronal UP states through a purinergically mediated mechanism. This coupled with the recent report that glutamate-stimulated glycogenolysis in astrocytes cause astrocytes to release ATP (a mediator of neuronal excitability via the K_ATP_ channel [130]) provides an increasingly rich picture of the deep relationship between network-level metabolism involving multiple cell types and the brain's intrinsic activity.

Finally, there is a long history in biochemistry of metabolic rhythms remarkably similar in character to the spontaneous fluctuations in the fMRI BOLD signal (for a comprehensive review of this fascinating work, see [131]), where glycolysis plays a central role. In a long overlooked work, it was noted that cellular redox states, a direct manifestation of metabolic activity, fluctuate synchronously in homologous regions of the hemispheres [132] in a manner not unlike that shown in figure 1*b*. More recent work has implicated changes in cellular redox states as critical for neuronal electrical function [133]. A noteworthy quote from the latter work is worth our consideration: ‘Energetic fluctuation in the central nervous system has been considered to be a consequence of neuronal activity. However, our study implies that changes in cellular metabolic state could be the *cause*, rather than the *result*, of neuronal activity’ [133, p. 842].

The way forward is clear; we must be open-minded when considering issues influencing brain function that can lead us to a better understanding of the brain's intrinsic activity.

## 7. Summary

There has been a long tradition in neuroscience of studying neuronal responses to stimuli and activity during task performance. In this work, the role of bottom–up and top–down (or feed forward or feedback) causality is frequently discussed, reflecting a debate that extends back at least a century on the relative importance of intrinsic versus evoked activity in brain function [36]. More recently brain imaging in humans has added a new dimension to this discussion both in terms of the large-scale organization of intrinsic activity and also its cost.

Presently, we know that intrinsic activity is a complex tapestry of highly interrelated activities across levels of analysis from behaviour and large-scale brain systems to cells, their membrane properties, metabolism and genes. In addition to the remarkable organization of this activity is the fact that it is largely responsible for the enormous cost of brain function. Together these facts have led to the growing realization that intrinsic activity is vitally important for brain function across the lifespan.

In achieving the goal of understanding intrinsic activity more fully, integrating information from multiple levels of analysis will be required. This will be challenging but ultimately rewarding in coming to a better understanding of the human brain in health and disease. The intellectual and societal rewards of embracing this challenge are well worth the effort.

## Endnotes

1 The idea of the brain's dark energy had its origins in my thinking when the large difference between the energy devoted to behaviourally evoked activity (very small) and that devoted to ongoing brain function (very large) became apparent to me. In selecting the term ‘dark energy’, I wanted to highlight the neglect that this discrepancy has endured and the lack of understanding that has resulted. Some will immediately note that I have unashamedly borrowed the term dark energy from cosmology where it has enjoyed a long history in discussions of the origin and evolution of the universe by calling attention to a major constituent of the universe (i.e. energy) that we have yet to fully understand. Viewed that way, it seemed to me that dark energy applied equally well to the brain in terms of its ongoing energy consumption.

2 The *resting state* is here viewed as a behavioural state characterized by quiet repose usually with eyes closed but occasionally, in the experimental setting, with eyes open with or without visual fixation (visual fixation as a resting state proxy probably only applies to humans where maintaining visual fixation is near effortless compared to monkeys who must be coerced). We presume that during the resting state subjects experience an ongoing state of conscious awareness largely filled with stimulus independent thoughts. It is important to distinguish between the resting state, defined behaviourally, and the state of the brain that accompanies the resting state which is one of high energy consumption that varies little between the resting state and engagement in attention demanding tasks.
